# Etymologia: *Pseudoterranova azarasi*

**DOI:** 10.3201/eid1709.110541

**Published:** 2011-09

**Authors:** Scott A. Norton, David I. Gibson

**Affiliations:** Author affiliations: Georgetown University, Washington, DC, USA (S.A. Norton);; Natural History Museum, London, UK (D.I. Gibson)

**Keywords:** etymology, Pseudoterranova azarasi, Ascaridida, Antarctic, Nematoda, taxonomy, history, ships, Robert F. Scott, Terra Nova expedition, letter

**To the Editor**: Regarding the March 2011 Etymologia on *Pseudoterranova azarasi* (*1*), we think that someone literally missed the boat on the derivation of *Pseudoterranova*. Although the Greco-Latin amalgam, *Pseudoterranova*, translates to “false new earth,” the generic name of the organism refers to the ship, the *Terra Nova*, which Robert Falcon Scott captained en route to Antarctica exactly 100 years ago in his ill-fated attempt to be the first person to reach the South Pole.

During the Antarctic summer of 1911–12, while Scott and 4 companions trudged toward the South Pole, the ship’s surgeon, Edward Leicester Atkinson, who remained with the *Terra Nova*, dissected polar fish, birds, and sea mammals, looking for parasites. Atkinson found an unusual nematode in a shark, and in 1914, he, along with parasitologist Robert Thomson Leiper of the London School of Tropical Medicine, commemorated the ship by conferring the name *Terranova antarctica* upon this newly discovered creature (*2*).

The genus *Pseudoterranova* was established by Aleksei Mozgovoi in 1951 for a somewhat similar nematode obtained from a pygmy sperm whale. *Pseudoterranova azarasi*, the subject of the Etymologia, was originally described in 1942 as *Porrocecum azarasi*, but recent molecular work, as described by Arizono et al. (*3*) and Mattiucci and Nascetti (*4*), showed that this nematode is part of a large species complex within *Pseudoterranova*. Thus, it has been transferred to this genus as part of the *P. decipiens* species complex.

The nomenclatural specifics are complex and arcane. However, in this centennial year of the *Terra Nova* expedition, we think it is worthwhile to remember the historic origins of these names.
